# Supplementary material for: The Systems Biology Research Tool: evolvable open-source software
Source: BMC Syst Biol. 2008 Jun 29;2:55. doi: 10.1186/1752-0509-2-55 (PMC2446383; doi:10.1186/1752-0509-2-55)
Supplement: Additional file 1 — SBRT Archive. An archive of the current version of the Systems Biology Research Tool. [file 1752-0509-2-55-S1.zip › sbrt-1.4.0/doc/developers_guide/api/sbrt/kernel/fba/comp/package-summary.html]

sbrt.kernel.fba.comp


|  |  |  |
| --- | --- | --- |
| **Overview** \| **Package** \| Class \| **Use** \| **Tree** \| **Deprecated** \| **Index** \| **Help** | |  |
| **PREV PACKAGE**   **NEXT PACKAGE** | **FRAMES**    **NO FRAMES**     **All Classes** |  |


---

## Package sbrt.kernel.fba.comp

Provides classes and interfaces for performing Flux Balance Analysis computations.

**See:**
  
          **Description**

| **Interface Summary** | |
| --- | --- |
| **CycleComputer** | This interface is used to represent algorithms for identifying steady state cycles (Type III extreme pathways) in stoichiometric networks. |
| **ExtremeCurrentComputer** | This interface is used to represent extreme current computers. |
| **FluxCvComputer** | This interface is used to represent a flux constraint variation computer. |
| **FluxIntervalComputer** | This interface is used to represent flux interval computers. |
| **FluxomeReducer<T extends Fluxome>** | This interface is used to represent algorithms that reduce the size of fluxomes. |

| **Class Summary** | |
| --- | --- |
| **AbstractCvComputer** | This abstract class is a skeleton implementation of `FluxCvComputer`. |
| **AbstractFluxIntervalComputer** | This abstract class is a skeleton implemenation of `FluxIntervalComputer`. |
| **CdHarFluxVectorComputer** | This class is used to compute randomly distributed vectors within flux space. |
| **FastMsFluxRangeComputer** | This class is used to compute Mahadevan-Schilling flux intervals in a (sometimes) faster way than `MsFluxIntervalComputer` can. |
| **FbaOptComputer** | This class is the workhorse of all Flux Balance Analysis optimizations. |
| **FbaSolver** | This class is used to solve the system of linear equations represented by *Sv = 0*. |
| **FluxCapComputer** | This class is used to compute *flux caps* for use in Mahadevan-Schilling flux interval computations. |
| **InitialPointComputer** | This class is used to compute initial points for coordinate-direction hit-and-run random flux vector algorithms. |
| **MsFluxIntervalComputer** | This class is used to compute Mahadevan-Schilling flux intervals. |
| **MsFluxomeReducer** | This class is used to reduce the size of fluxomes for the purpose of identifying the steady state cycles (Type III extreme pathways) they contain. |
| **ReverseRxnFluxIntervalComputer** | This class is used to compute flux intervals while constraining the fluxes of reverse reactions to their minimum. |
| **RxnDeletionComputer** | This class is used to compute the effect of deleting reactions in a fluxome. |
| **SimpleFluxIntervalComputer** | This class is used to compute flux intervals in the simplest possible way. |
| **SlpCycleComputer** | This class is used to identify the steady state cycles (Type III extreme pathways) in a fluxome. |
| **SlpExCurrComputerV2** | This class is an implementation of an extreme current algorithm. |
| **WwFluxomeReducer** | This class is used to reduce the size of fluxomes for the purpose of identifying the steady state cycles (Type III extreme pathways) they contain. |

| **Exception Summary** | |
| --- | --- |
| **FbaOptException** | This class is used to indicate that an optimal solution could not be found or does not exist. |
| **FluxCapException** | This class is used to indicate that a flux cap is invalid. |
| **FluxRangeException** | This class is used to indicate that the range of flux interval has exceeded a defined cutoff value. |
| **InvalidFluxVectorException** | This class is used to indicate that a flux vector is invalid. |

## Package sbrt.kernel.fba.comp Description

Provides classes and interfaces for performing Flux Balance Analysis computations.

---


|  |  |  |
| --- | --- | --- |
| **Overview** \| **Package** \| Class \| **Use** \| **Tree** \| **Deprecated** \| **Index** \| **Help** | |  |
| **PREV PACKAGE**   **NEXT PACKAGE** | **FRAMES**    **NO FRAMES**     **All Classes** |  |


---
